# Impact of Tibial Component Coronal Alignment on Knee Joint Biomechanics Following Fixed‐bearing Unicompartmental Knee Arthroplasty: A Finite Element Analysis

**DOI:** 10.1111/os.12927

**Published:** 2021-05-20

**Authors:** Yong Nie, Qiang Yu, Bin Shen

**Affiliations:** ^1^ Department of Orthopaedic Surgery and Orthopaedic Research Institute and National Clinical Research Center for Geriatrics West China Hospital, West China Medical School, Sichuan University Chengdu China; ^2^ Department of Orthopaedic Surgery Chengdu 7th people's Hospital Chengdu China

**Keywords:** Biomechanics, finite element analysis, Fixed‐bearing, Unicompartmental knee arthroplasty

## Abstract

**Objective:**

Unicompartmental knee arthroplasty (UKA) has indicated a higher rate of revision than total knee arthroplasty (TKA). The success of UKA depends on UKA component alignment, fixation, and soft tissue integrity. The purpose of this study was to investigate the effects of different tibial component alignments in the coronal plane on the stress distribution in UKA. It was hypothesized that the stress distribution would approach native knee when the tibial component was neutrally positioned.

**Methods:**

The left legs from two healthy volunteers were considered to represent the geometric native knee models. All bones within the knee joint were extracted from the three‐dimensional (3D) computed tomography (CT). MRI was used to generate cartilage, menisci, and four major ligaments. The UKA components were virtually implanted in the medial compartment of the knee model using MIMICS. A total of five different configurations of UKA tibial obliquity in the coronal plane (neutral, 3° varus, 6° varus, 3° valgus, and 6° valgus) were adopted and investigated. Subject‐specific inhomogeneous material properties of bones were used in the finite element analysis (FEA) model. The von Mises stress in the tibia platform and proximal tibia, and the load distribution between the medial and lateral compartments were extracted and compared among the five different configurations.

**Results:**

The inhomogeneous material properties of the trabecular bone were closer to real physics than traditional homogeneous methods. Neutral and 3° varus alignments of the tibial component in the coronal plane have better stress distribution between medial and lateral compartment as healthy knee models, and less stress‐shielding effects than other UKA configurations. The stress pathway under the medial tibia platform in neutral and 3° varus UKA configurations was similar and more obvious than the other three UKA configurations. Notably, the stress of the medial tibia platform in the 3° varus UKA models was more homogenous than the neutral UKA configuration. The 6° varus, 3° valgus, and 6° valgus UKA models had higher stress at the location of anterolateral and posterolateral tibia platform than other UKA configurations.

**Conclusion:**

Neutral or 3° varus positioned in the coronal plane for the tibial component could be the optimal alignment for UKA. Excessive varus or valgus obliquity in the coronal plane lead to significant differences in bone stress transfer and load distribution in the knee, and increase the risk of UKA failure.

## Graphical abstract

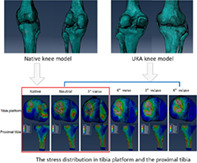

## Introduction

Knee osteoarthritis (OA) is a major cause of pain and physical disability. It is estimated that 6% of those aged 30 years and older and 15% of those aged 45 years and older experience the condition, with a lifetime risk of 45%^1^. The compartments of the knee are not all affected in the same way; people are at 10 times greater risk of having medial than lateral knee OA. In the 1950s, McIntosh and Hunter first used a metal spacer in single tibiofemoral compartment for painful varus and valgus deformities. In the 1960s and 1970s, the St Georg and Marmor prostheses showed good outcomes for unicompartmental OA^1^. Both of these designs had polycentric metal femoral condyles that articulated on flat, fixed polyethylene tibial components, with the femoral and tibial components cemented to the bone. In 1974, the first mobile‐bearing unicompartmental knee arthroplasty (UKA) was introduced and was first reported in 1988^1^.

UKA is a surgical procedure used to relieve arthritis in one of the knee compartments in which the damaged parts of the knee are replaced. UKA has gained interest in recent years because it can diminish postoperative pain and therefore is an effective treatment for localized osteoarthritis of the knee. UKA has demonstrated exceptional success rates in several studies^2^. Since the integrity of the anterior cruciate ligament, and lateral and patellofemoral compartments, are preserved in the UKA, the kinematics of the knee after UKA is similar to native knee^3^. Furthermore, UKA is a less invasive procedure that provides faster recovery and less blood loss than total knee arthroplasty (TKA)^4^. Although patients received UKA had a higher satisfaction rate than TKA, the survival rate of UKA was worse than TKA^5^. UKA may fail for many reasons. The major reasons are the implant type used, unexplained pain, surgeon's skill, age, body weight, alignment, and implant position^5^.

The coronal malalignment of the tibial component is considered as a significant factor related to UKA failure because of abnormal stress and OA progression of the contralateral compartment^5^. However, the optimal alignment of the tibial component remains unclear. Previous studies showed that the valgus alignment of the tibial component in the coronal plane and higher posterior slope in the sagittal plane should be avoided in terms of the survival rate of the implant^6^. Sekiguchi *et al*.^6^ suggested that the preferred tibial component alignment is between neutral and 2° varus in the coronal plane. Varus >4° or valgus alignment caused excessive Medial/lateral (ML) translation, which could be related to feelings of instability and could potentially have negative effects on clinical outcomes and implant durability^6^. Recently, Asada *et al*.^5^ indicated that the tibial component should be installed 4° to 2° varus to the tibial mechanical axis to maintain joint‐line parallelism for UKA in medial osteoarthritis patients. Barbadoro *et al*. used the maximal total point motion (MTPM) as a predictor for implant loosening, and reported that there is correlation between varus orientation of the tibial component and MTPM from radiostereometry in UKA. Particularly, a misalignment in varus larger than 5° could lead to risk of loosening the tibial component^7^.

In addition, to investigate the effects of different tibial component alignments on the stress distribution in UKA, previous studies utilized finite element analysis (FEA) and evaluated the effects on tibia stress^8^, ^9^, ^10^. Zhu *et al*.^11^ showed that tibial component coronal alignment can greatly affect the static knee biomechanics after UKA. They recommended a range from 4° valgus to 4° varus inclination of tibial component in mobile‐bearing UKA^11^. However, Iesaka *et al*. suggested that slight valgus inclination of the tibial component might be preferable to varus and even to 0° inclination so far as the stress distribution is concerned^12^. These results showed that the optimal alignment of the tibial component in UKA still remains a matter of controversy. Moreover, it should be noted that the FEA models used in those previous studies are not subject‐specific, and the material properties of the cortical and trabecular bone are considered as homogenous. Thus, the stress pathway in the proximal tibia may not reflect the complex stress distribution in the trabecular bone and cause bias.

Despite the newer advancements over past decades and proven advantages of minimally invasive UKA, the effects of the alignment of the tibial component on stress in UKA remain unclear. The aims of this study were to: (i) use subject‐specific FEA models for stress analysis in the knee joint after UKA; (ii) investigate the effects of different tibial component alignments in the coronal plane on the knee stress after UKA; (iii) compare the stress distribution and stress pathway between the media and lateral in proximal tibia. It was hypothesized that the stress distribution would approach native knee when the tibial component was neutrally positioned.

## Materials and Methods

### Native Knee Model

**Fig. 1:**
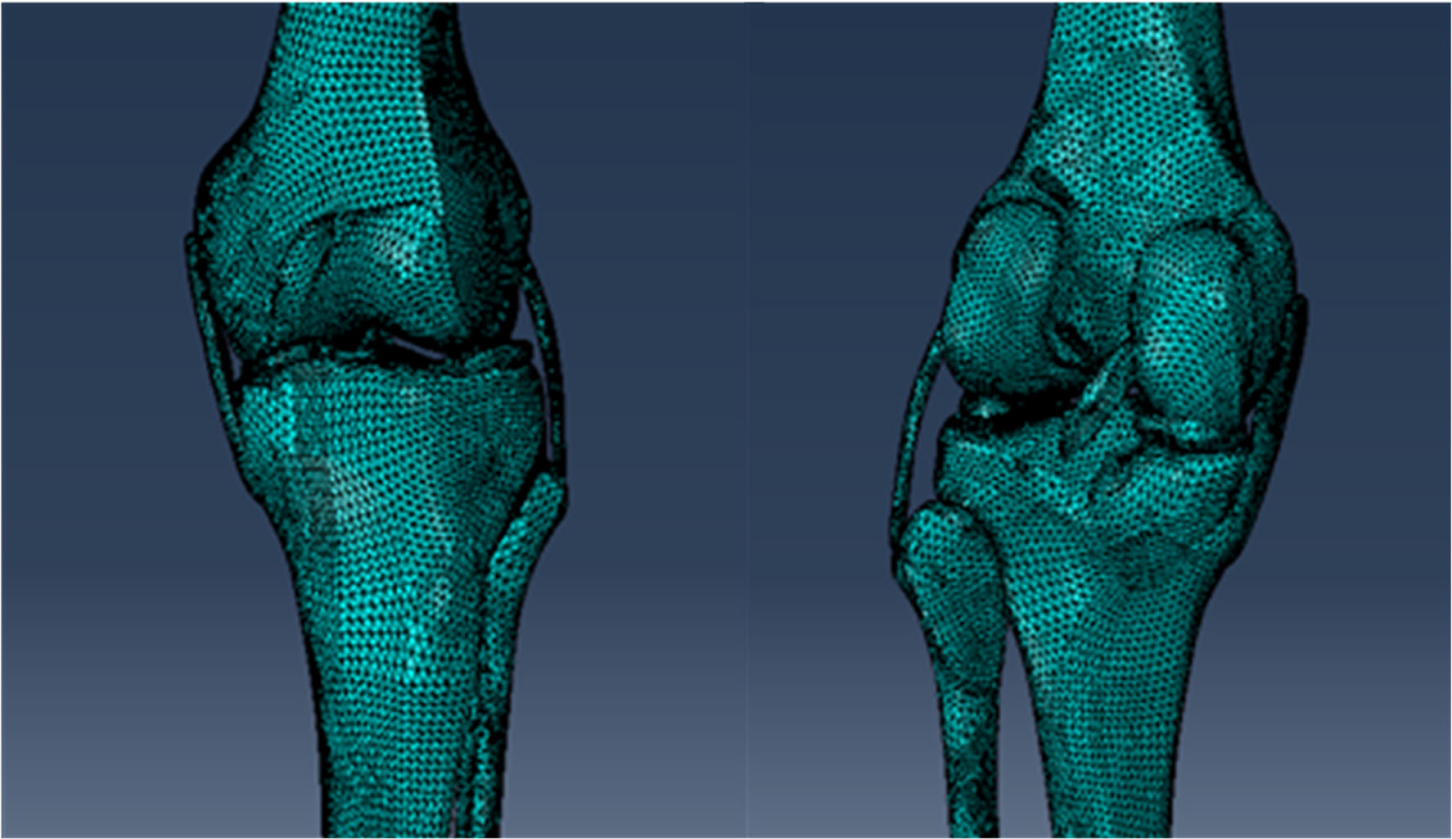
Native knee model.

The left legs from a 36‐year‐old man (with a height of 170 cm and a bodyweight of 65 kg) and a 30‐year‐old man (with a height of 165 cm and a bodyweight of 60 kg) was considered to represent the geometric native knee models. Informed written consent was obtained from participants in this study. All bones within the knee joint (i.e. femur, tibia, and fibula) were extracted from the 3D CT. MRI was used to generate cartilage, menisci, and four major ligaments: lateral collateral ligament, medial collateral ligament, anterior cruciate ligament, and the posterior cruciate ligament. The surface geometries of volume were extracted from CT and MRI *via* isosurfacing and image‐based thresholding, as implemented in MIMICS (version 10.01, Materialise NV, Leuven, Belgium). The ensuing triangulated surface data were exported in the stereolithography (STL) format for post‐processing in GeomagicStudio (version 12.0.0, Geomagic, North Carolina, USA)^13^. The solid model of the knee joint was then constructed (Fig. 1).

**TABLE 1:** Material properties assignment in UKA FEA model

Materials	Elastic modulus (MPa)	Poisson's ratio
Cortical bone	17,000	0.30
Trabecular bone	Inhomogeneous	0.20
Cartilage	15	0.46
Menisci	27.5	0.33
ACL	169	0.45
PCL	177	0.45
MCL	332	0.45
LCL	345	0.45
Titanium (Tibial component)	11,000	0.34
CoCrMo alloy (Femoral component)	210,000	0.29
UHMWPE	850	0.40

ACL, anterior cruciate ligament; CoCrMo alloy, Cobalt‐chromium‐molybdenum alloy; LCL, lateral collateral ligament; MCL, medial collateral ligament; PCL, posterior cruciate ligament; UHMWPE, Ultrahigh‐molecular‐weight‐polyethylene.

### UKA Knee Model

**Fig. 2:**
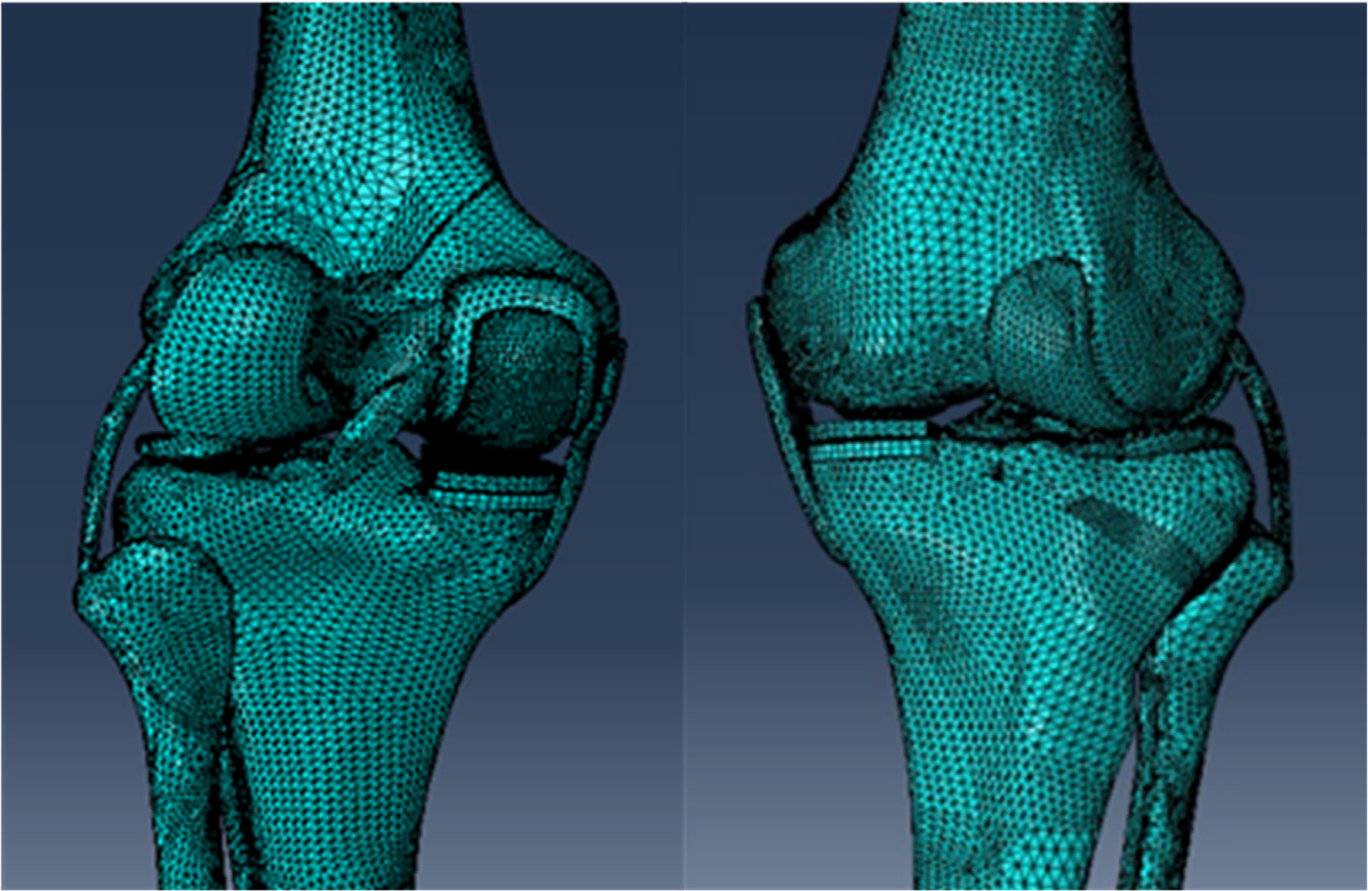
UKA knee model.

Once the native knee model was developed, a fixed bearing UKA (Zimmer Inc., Warsaw, Indiana, USA) was selected. The 3D model in the STL format of UKA components was obtained by using the 3D scanner (Open Technologies, Italy). The UKA components were virtually implanted in the medial compartment of the knee model in MIMICS (version 10.01, Materialise NV, Leuven, Belgium). A total of five different configurations of UKA tibial component obliquity in the coronal plane were simulated and investigated. The neutral position was defined as cutting the tibia orthogonal to the coronal tibial mechanical axis (Fig. 2). Varus/valgus positions of 3° and 6° were achieved by an equivalent repositioning of the tibial component from the neutral position.

### Subject‐specific Inhomogeneous FEA Model

Linear, four‐node tetrahedral elements were chosen for all tissues in Abaqus 6.10.1 (Simulia, Rhode Island, USA)^14^. Based on the mesh convergence analysis results, 2 mm element edge lengths were used for all bones and 1 mm for cartilage, meniscus, and ligaments. Subject‐specific material properties of bones were adopted. The cortical bone was represented with a single isotropic elastic modulus of 17,000 MPa^15^. For trabecular bone, the nonlinear, apparent density–elastic modulus relationship (Elastic modulus = 8920 × (ρ_apparent density_)^1.83^) developed by Morgan^16^ was used to assign material properties for each element. Poisson's ratio of 0.3 and 0.2 was assigned for cortical and trabecular bone elements, respectively. The menisci and cartilage were modeled as linear‐elastic materials due to the incompressible nature of cartilage tissue under short loading times. Interfaces between the cartilage and bones were modeled as fully bonded. Both menisci were attached to the tibia at the horns, and the medial meniscus was also attached along the peripheral edge^2^. Small sliding interactions with zero friction were defined between the femoral and tibial cartilage on the lateral and medial sides. Ligament models were considered isotropic and hyper‐elastic materials to represent their nonlinear stress–strain relations.

In the simulation, the distal fibula and tibia were constrained in all degrees of freedom, while the femur was completely unconstrained except for the flexion angle, which was fixed in full extension. A single‐leg stance was assessed for model simplification and focused on the joint stress under a maximum physiologic load condition during gait. A vertical load (N), equal to bodyweight (kg) × 10 (m/s^2^), was applied to the proximal femur for each patient^13^. The subject‐specific FEA model was validated in a previous study^17^.

For all the developed models (the native knee and the five replaced models), the von Mises stress in the proximal tibia and the load distribution between the medial and lateral compartments were extracted and compared among the different configurations. Meanwhile, the stress values in the region of interest (ROI) (anteromedial, posteromedial, anterolateral, and posterolateral) of the tibia platform were extracted and assessed.

## Results

### Stress Distribution in the Proximal Tibia

**Fig. 3:**
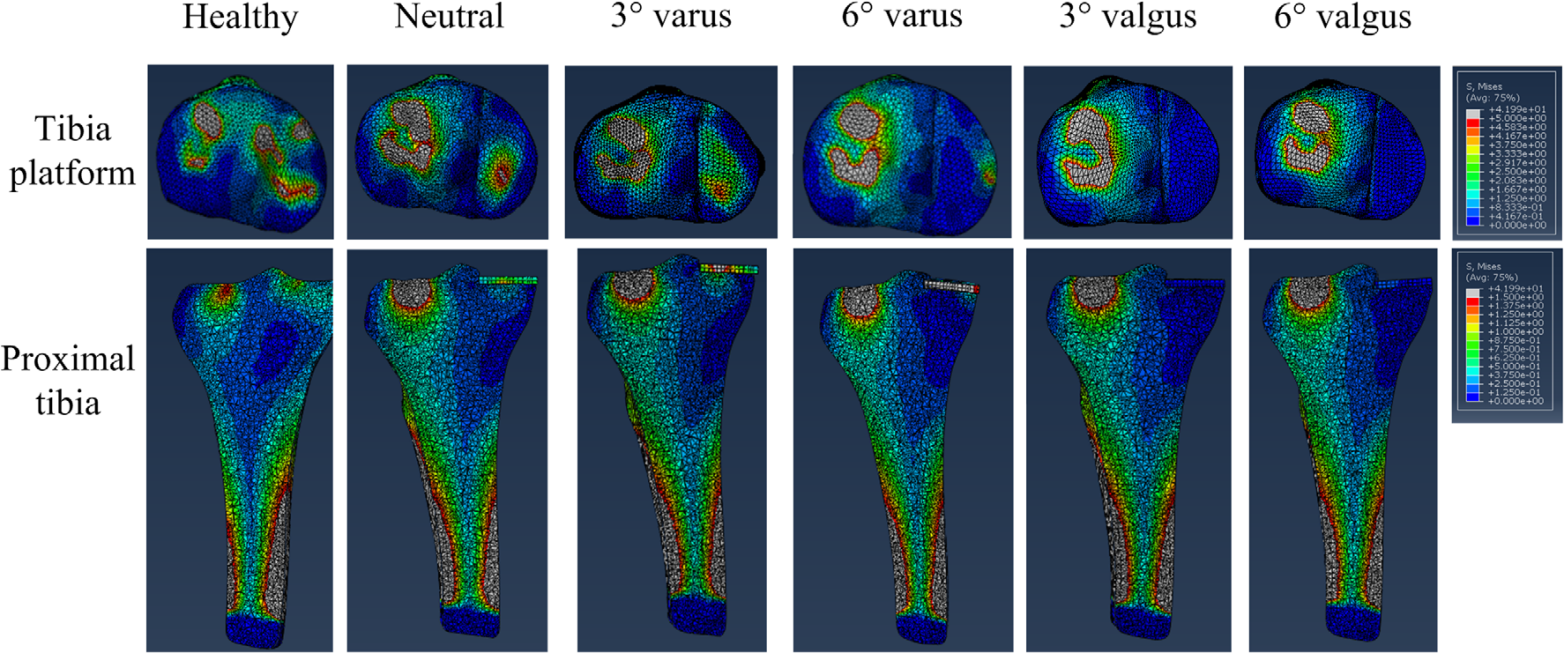
The von Mises stress distribution on tibia platform and in the proximal tibia (of a 36‐year‐old man with a height of 170 cm and a bodyweight of 65 kg).

**Fig. 4:**
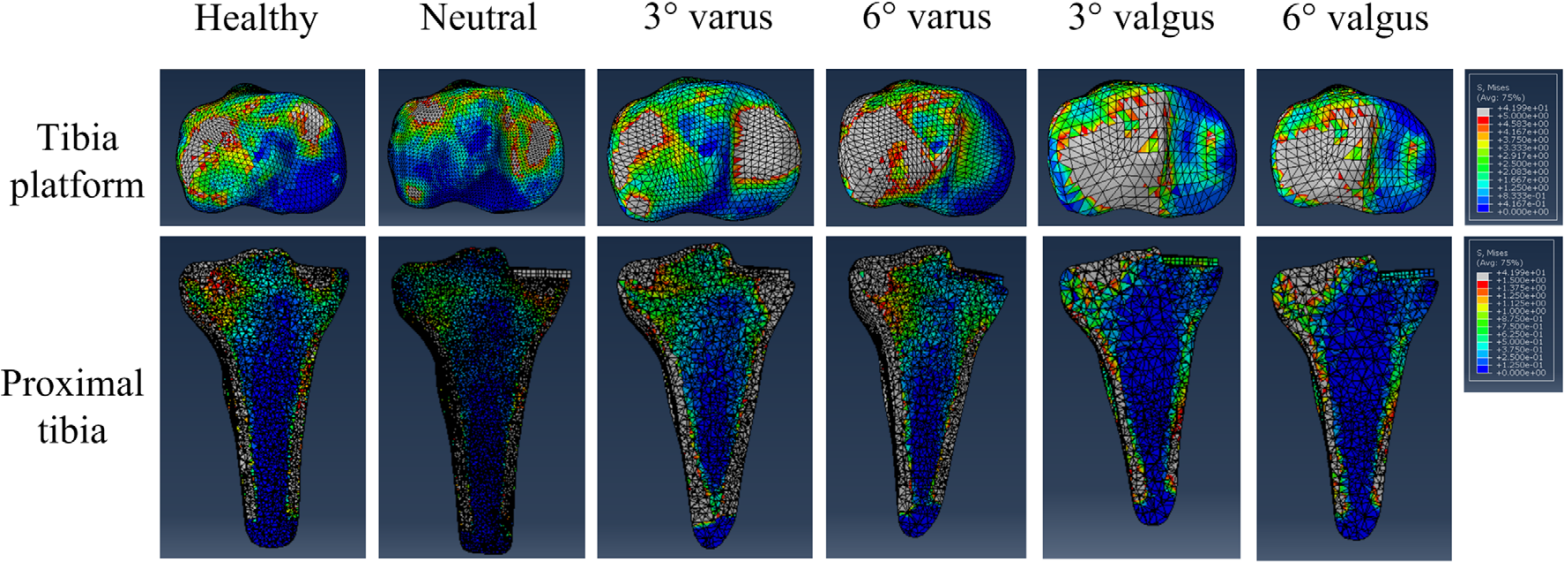
The von Mises stress distribution on tibia platform and in the proximal tibia (of a 30‐year‐old man with a height of 165 cm and a bodyweight of 60 kg).

In the native (healthy) models, the stress distribution was more symmetrical between the medial and lateral tibia platforms than all five UKA configurations. The neutral and 3° varus UKA models had more stress distribution in medial tibia platform than the other three UKA configurations (6° varus, 3° valgus, and 6° valgus). Notably, the stress of the medial tibia platform in the 3° varus UKA models was more homogenous than the neutral UKA configuration. Besides, all five UKA configurations had similar stress distribution in the lateral tibia platform (Figs 3, 4).

The UKA models had more stress transfer under the lateral tibia platform but less under the medial tibia platform than the native (healthy) model. The stress pathway under the medial tibia platform in neutral and 3° varus UKA configurations was similar and more obvious than the other three UKA configurations (Figs 3, 4).

### Stress Value in the ROI

**Fig. 5:**
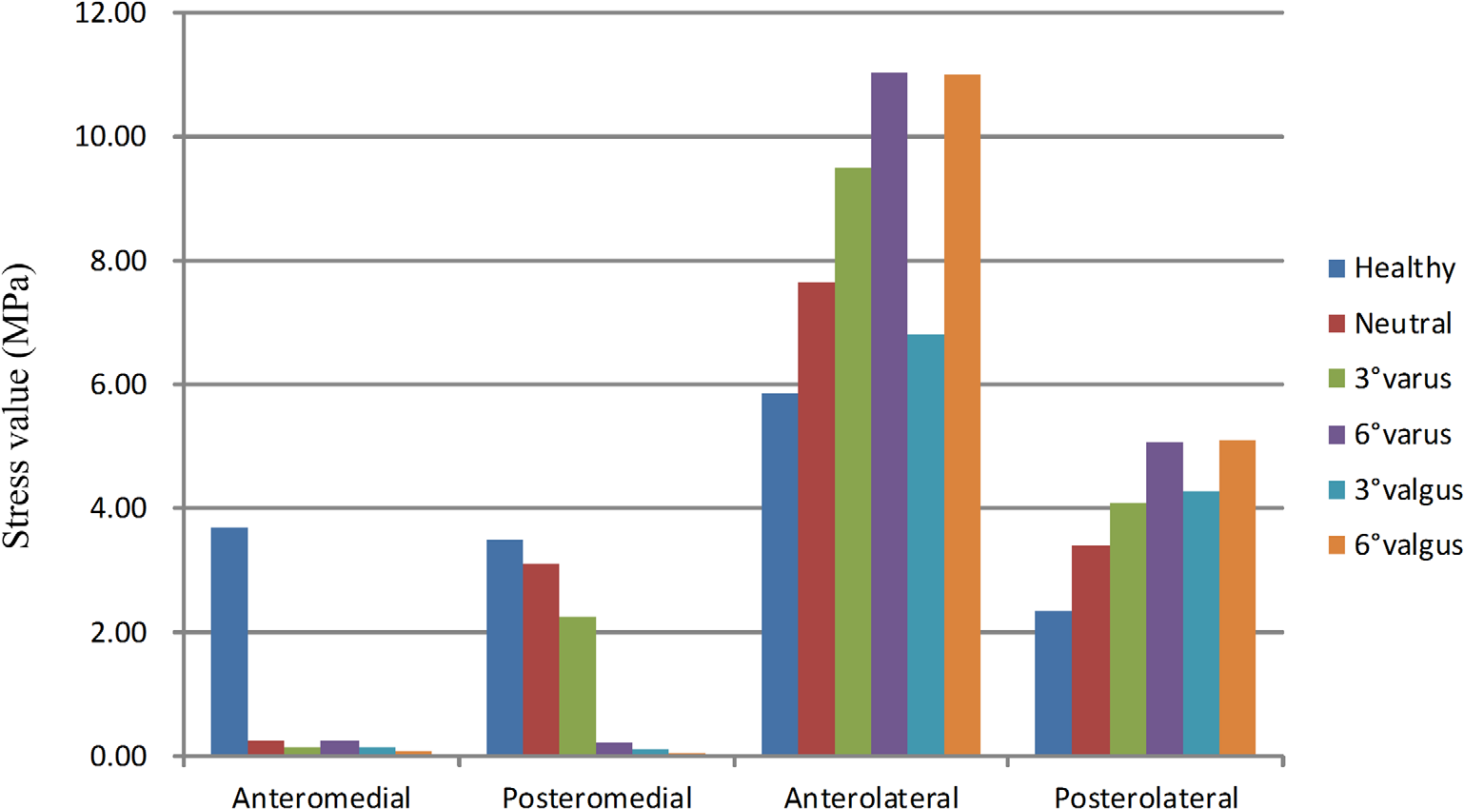
Mean stress value in the region of interest (of a 36‐year‐old man with a height of 170 cm and a bodyweight of 65 kg).

**Fig. 6:**
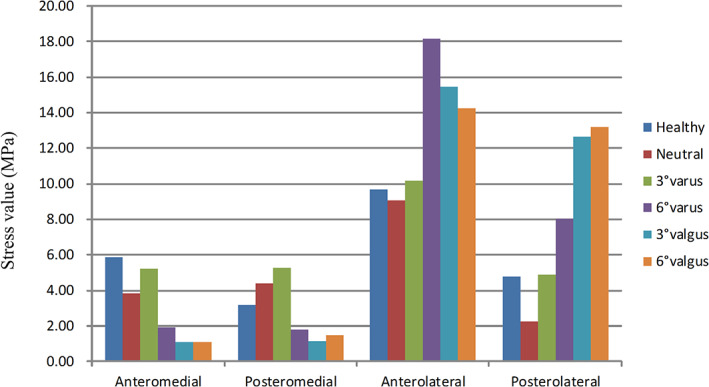
Mean stress value in the region of interest (of a 30‐year‐old man with a height of 165 cm and a bodyweight of 60 kg).

At the location of the anteromedial tibia platform (ROI‐1), the mean stress values in UKA configurations were lower than the native (healthy) model. At the location of the posteromedial tibia platform (ROI‐2), the mean stress values in neutral and 3° varus UKA configurations were closer to the native model than the other three UKA configurations. In addition, the 6° varus, 3° valgus, and 6° valgus UKA models had higher mean stress value at the location of anterolateral (ROI‐3) and posterolateral tibia platform (ROI‐4) than other UKA configurations (Figs 5, 6).

## Discussion

The study aimed at to investigate the effects of different tibial component coronal plane alignments of UKA on the stress in the knee using a subject‐specific FEA model. The optimal tibia alignment for UKA was also assessed. Results of this study showed that erroneous varus or valgus obliquity in the coronal plane lead to significant differences in bone stress and load distribution in the knee. Neutral or 3° varus in the coronal plane for the tibial component could be the optimal alignment for UKA.

Most of the FEA models for knee joint adopted a homogenous material assignment and different elastic modulus values for cortical and trabecular bone. Innocenti *et al*.^8^ assigned the trabecular bone with the elastic modulus of 2130 MPa and 0.31 Poisson's ratio to generate the UKA FEA model. Wen *et al*.^18^ used the elastic modulus of 350 MPa and Poisson's ratio of 0.25 to represent the material properties of the trabecular bone. Inoue *et al*.^9^ applied a stiffness of 0.83 GPa to metaphyseal trabecular bone and 13.4 GPa to the cortical bone. In these FEA studies for UKA, the material properties assignments did not reflect the site‐specific properties in different regions of the bone and thus could cause bias in investigating the stress distribution and mean stress value at local and especially at different regions of the trabecular bone. In our study for the trabecular bone, the nonlinear, apparent density–elastic modulus relationship (Elastic modulus = 8920 × [ρ_apparent density_]^.83^) developed by Morgan^16^ was used for each element. With this method, the different trabecular bone sites had different material properties. The material properties of the trabecular bone were inhomogeneous and closer to real physics. Therefore, this subject‐specific FEA model is more suitable for stress analysis at local and different regions of the trabecular bone in UKA (Table 1).

As for the coronal plane alignment of the tibial component in UKA, previous studies reported that excessive varus alignment could worsen the survivorship of UKA and increase the risk of loosening^6^. The biomechanical effects of different coronal plane alignment of the tibial component on stress change were recently analyzed using FEA models. Inoue *et al*.^9^ demonstrated that the valgus obliquity of the tibial component remarkably increased the stress concentration on the medial tibial metaphyseal cortex and the posterior tibial cortex. Placement of the tibial component using a large valgus obliquity may increase the risk of medial tibial condylar fractures. Innocenti *et al*.^8^ recommended neutral tibial alignment or a slight varus alignment (3°) based on collateral ligament strain and bone and polyethylene insert stress distribution. Sekiguchi *et al*.^6^ suggested that the preferred tibial component alignment is between neutral and 2° varus in the coronal plane. Varus >4° or valgus alignment caused excessive medial/lateral translation, which could be related to feelings of instability and could have negative effects on clinical outcomes and implant durability. In this present study, neutral and 3° varus alignments were preferred according to the stress distribution in the proximal tibia and the stress pathway under the tibia platform compared with the native knee joint. From a subject‐specific stand point, our study strengthens previous clinical findings and biomechanical theories.

Aseptic loosening of the UKA components, and especially of the tibia, is one of the main failure modes in UKA^8^. Excessive stresses cause it in both cortical and trabecular bone, which lead to stress shielding. In our study, the stress distribution in the proximal tibia has changed after the implantation of UKA components. Compared with the symmetrical load between medial and lateral tibia in the native knee, all configurations of UKA had more load transfer in proximal lateral tibia than medial tibia. This is mainly caused by the change in stiffness between the medial and the lateral compartments induced in the knee by the UKA components^8^. In contrast to the native lateral compartment, the medial UKA components have larger elastic modulus. Thereby, the stress in the trabecular bone underneath the tibial component dropped sharply compared with the native knee model. However, our findings indicate that neutral and 3° varus alignments of the tibial component in the coronal plane have better stress distribution between the medial and lateral compartments, and less stress shielding effect than other three UKA configurations (6° varus, 3° valgus, and 6° valgus).

Finally, we would like to mention some limitations of this present study. Only two subjects’ knee joints and only the fixed bearing UKA components have been included and virtually implanted. Besides, the position of the femoral component was not investigated. Future studies investigating the effects of both the femoral and tibial component malalignment on the stress distribution and loading transfer in the proximal tibia would provide further information to explain the clinical outcomes and predict the risk of implant durability.

### Conclusion

Neutral or 3° varus in the coronal plane for the tibial component could be the optimal alignment for UKA. Excessive varus or valgus obliquity in the coronal plane lead to significant differences in bone stress transfer and load distribution in the knee and increase the risk of UKA failure.
